# An Intraoperative Method to Minimize Leg Length Discrepancy in Anterior Minimally Invasive Total Hip Arthroplasty—A Prospective Study

**DOI:** 10.3390/jpm14060573

**Published:** 2024-05-27

**Authors:** Mauro Girolami, Roberto Bevoni, Elena Artioli, Renata Beluzzi, Cosimo Vasco, Silvio Caravelli, Annalisa Baiardi, Massimiliano Mosca

**Affiliations:** 1 Orthopaedic Department, IRCCS Istituto Ortopedico Rizzoli, 40010 Bentivoglio, Italy; 2 IRCCS Istituto Ortopedico Rizzoli, 40136 Bologna, Italy; 3 Department of Biomedical and Neuromotor Sciences (DIBINEM), Alma Mater Studiorum University of Bologna, 40123 Bologna, Italy; 4 Ospedale di Santa Maria della Scaletta, 40026 Imola, Italy

**Keywords:** total hip arthroplasty, leg length discrepancy, anterior minimally invasive surgery, compass, intraoperative measurements

## Abstract

While several intraoperative devices have been described in the literature for assessing leg length discrepancy (LLD), none have been utilized during total hip arthroplasty (THA) performed via the Anterior Minimally Invasive Surgery (AMIS) approach. The aim of this prospective study was to evaluate the efficacy and accuracy of a compass device in assessing leg length during THA performed using the AMIS technique. A prospective study was conducted involving 35 patients who consecutively underwent unilateral primary THA using the AMIS technique at our department from September 2017 to December 2018. LLD was measured by comparing preoperative and postoperative anteroposterior radiographs of the pelvis, independently assessed by two observers. The mean preoperative LLD was 3.6 (SD 3.9, range, 0.2–19.3) mm. The mean postoperative LLD was 2.5 (SD 3.0, range, 0–12.2) mm. A postoperative LLD of less than 5 mm was observed in 88.2% of cases, with 94.1% having values less than 10 mm. In conclusion, the compass device emerged as a valuable tool for ensuring precise limb length control in THA with the AMIS approach, offering both efficiency and cost-effectiveness in clinical practice.

## 1. Introduction

Primary total hip arthroplasty (THA) is a well-established orthopedic procedure that reliably provides pain relief and significant improvement of hip joint biomechanics. The goals of the surgery include restoration of the center of rotation, correct orientation of components, appropriate femoral offset, and equal leg length [1]. Among these key components of successful primary THA, leg length equalization still remains one of the most challenging targets [2,3]. Indeed, lengthening is a rather common occurrence after primary THA, with reported incidences ranging from 1% to 27% [2]. Leg Length Discrepancy (LLD) is a prevalent source of post-THA dissatisfaction [4] with patient frequently reporting mechanical symptoms, such as gait disturbances [5], hip instability potentially resulting in dislocations, and premature component loosening [2]. While uncommon, neurological sequelae have also been documented [6]. Consequently, LLD stands out as a leading cause of legal action [7,8]. A definitive threshold distinguishing acceptable from unacceptable LLD remains elusive [4], although numerous studies suggested that differences within 10 mm may be well tolerable [4,6,9]. 

**Figure 1:**
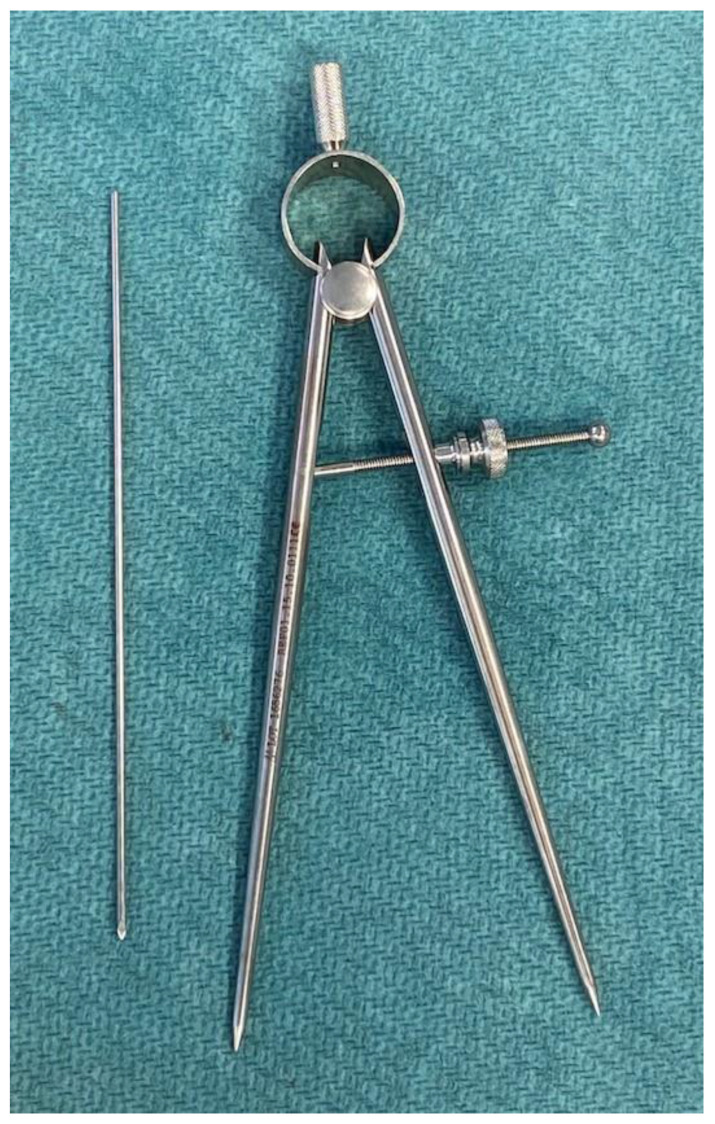
The compass device used for the measurements (MEDACTA International SA, Strada Regina, 6874 Castel San Pietro (CH)). The compass is long 200 mm and consists of two hinged arms with a central pivot point. One arm, equipped with a pointed end, is positioned at a specific location. By rotating the screw, the opening and closing of the other arm are enabled until reaching the second point of interest. The screw allows for maintaining the desired opening even after removing the compass, which can then be placed on a ruler to measure the desired distance (max 110 mm).

Several techniques have been outlined for assessing intraoperative leg length to mitigate LLD [10,11,12,13,14,15,16,17,18,19], yet none have been applied in minimally invasive anterior THA procedures. The aim of this study was to evaluate the efficacy and accuracy of a compass device (Figure 1) used for leg length assessment during minimally invasive anterior THA, with the goal of minimizing LLD and eliminating the necessity for intraoperative fluoroscopy.

The accuracy of the proposed method was compared against measurements obtained from pre- and post-operative radiographs. Additionally, a comprehensive literature review was conducted on strategies to prevent LLD in anterior THA, highlighting the advantages and drawbacks in comparison to the presented technique.

## 2. Materials and Methods

This prospective study received approval from the local Institutional Review Board (IRB) before enrollment. Detailed information was provided to the patients, and written consent was obtained prior to enrollment.

### 2.1. Patients Population

Starting from September 2017, patients aged 18 to 80 years with unilateral degenerative hip osteoarthritis suitable to primary THA via the anterior minimally invasive approach were consecutively recruited for this prospective study.

Exclusion criteria included previous hip fracture, contralateral hip arthroplasty, radiographic evidence of residual hip dysplasia, necessity for acetabular augmentation, cerebral palsy, or connective tissue disorders. 

A thorough preoperative clinical examination was conducted on all patients to assess the presence of preoperative LLD and to differentiate between radiological and functional causes (such as pelvic obliquity or muscular contracture). In addition, any lumbar scoliosis, sacroiliac joint issues, and pelvic obliquity were routinely assessed. Furthermore, understanding the patient’s perception was deemed crucial. The analysis of all these factors also contributed to assessing the risk of clinically noticeable postoperative LLD [20].

Each patient underwent pre-operative standing radiograph of the pelvis the day before surgery, which were repeated at the 3-month follow-up to measure pre- and post-operative leg length.

In instances where clinical and radiographic assessments concurred on LLD, the discrepancy was likely attributed to an anatomical factor necessitating intraoperative intervention. Conversely, when there was a discrepancy between clinical and radiographic findings, a functional component was indicated, which may be addressed through pre- and postoperative physiotherapy interventions [20].

All THAs were performed by the same experienced surgeon using the Anterior Minimally Invasive Surgery (AMIS) technique as described by F. Laude [21]. 

Patients were mobilized on the first post-operative day following a standardized rehabilitation protocol.

### 2.2. Compass Device

The instrument employed for the measurements was a compass device manufactured by MEDACTA International SA (Strada Regina, 6874 Castel San Pietro, Switzerland). This device had a length of 200 mm and consisted of two hinged arms connected at a central pivot point. One arm, featuring a pointed end, was positioned at a predefined location. The rotation of a screw enabled the opening and closing of the other arm until it reached the second point of interest. This screw mechanism allowed the compass to maintain the set opening even after it was removed from the measurement site. Subsequently, the device could be placed on a ruler to ascertain the measured distance. The minimum measurable distance was 10 mm, while the maximum was 110 mm.

### 2.3. Surgical Technique

The patient was positioned in supine decubitus with the affected leg placed on the AMIS Mobile Leg Positioner (Medacta International, Frauenfeld, Switzerland which was manipulated from outside the surgical field by an expert Product Specialist. The AMIS approach was executed between the tensor fasciae latae muscle laterally and the sartorius and rectus femoris muscles medially. The ascending branch of the lateral circumflex artery was isolated, ligated, and interrupted. Following incision of the joint capsule and removal of the periacetabular osteophytes, the most superior and lateral aspect of the acetabulum was exposed and marked with electric cautery. 

**Figure 2:**
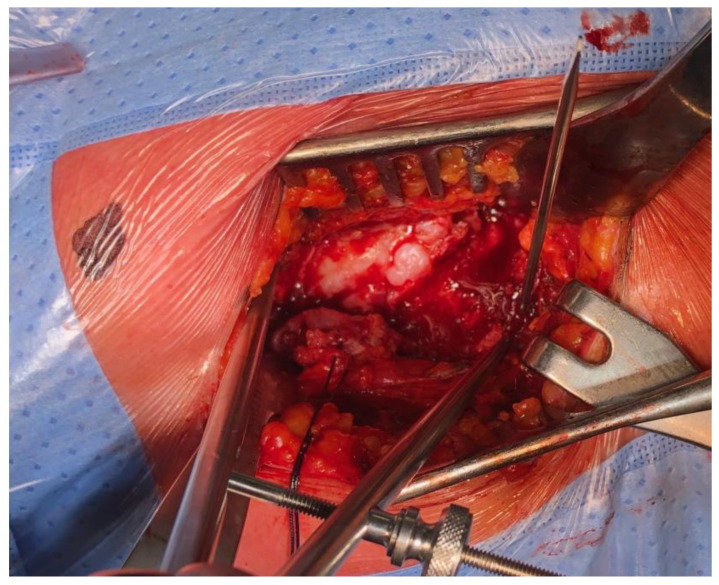
Measurement of the distance between the fixed acetabular and femoral marker, before the femoral neck osteotomy.

Before dislocation, without applying any traction, the affected leg was placed in a neutral position between extension and flexion and at 10 degrees of internal rotation using the Leg Positioner. A unicortical 2.0 mm K wire was then inserted at a point on the intertrochanteric line, as vertically as possible with respect to the mark on the acetabulum. The distance between these two landmarks was measured using the compass device (Figure 2). The K wires were subsequently removed to avoid hindering the preparation and placement of the implant, and their entry point was marked with an electrocautery device to facilitate their retrieval for subsequent measurements.

**Figure 3:**
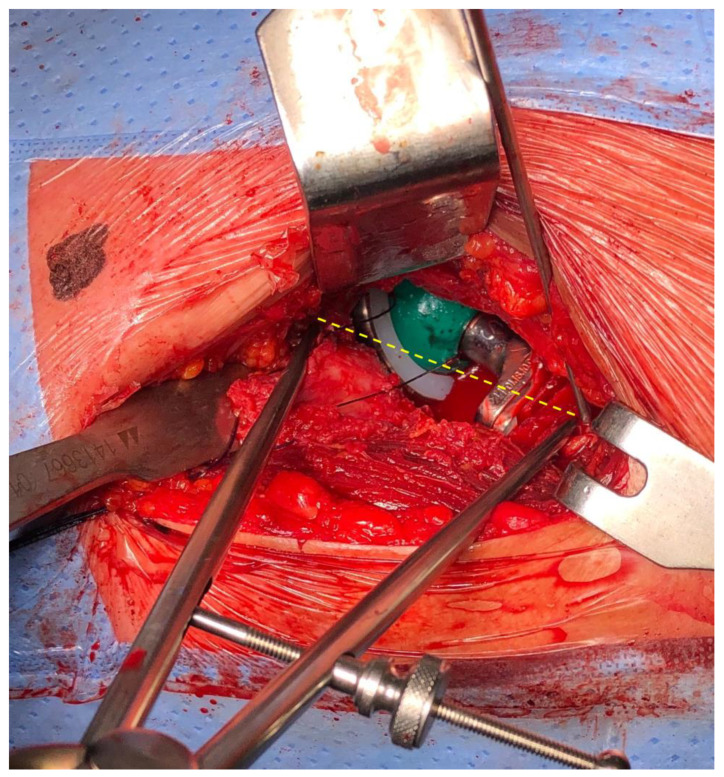
Measurement of the distance between the fixed acetabular and femoral marker (yellow line), with trial components.

A standard technique was employed to implant the uncemented acetabular cup (Versafit, Medacta International, Castel San Pietro, Switzerland). The choice between a Ceramic insert Allumina BIODEX or an Ultra-High Molecular Weight Polyethylene insert (Medacta International, Castel San Pietro, Switzerland) was made for each patient based on their age. Following the implantation of the femoral trial components, the hip was reduced, traction was released, stability was assessed, and leg length was measured using the compass device, following the previously described positioning of the leg (Figure 3). 

**Figure 4:**
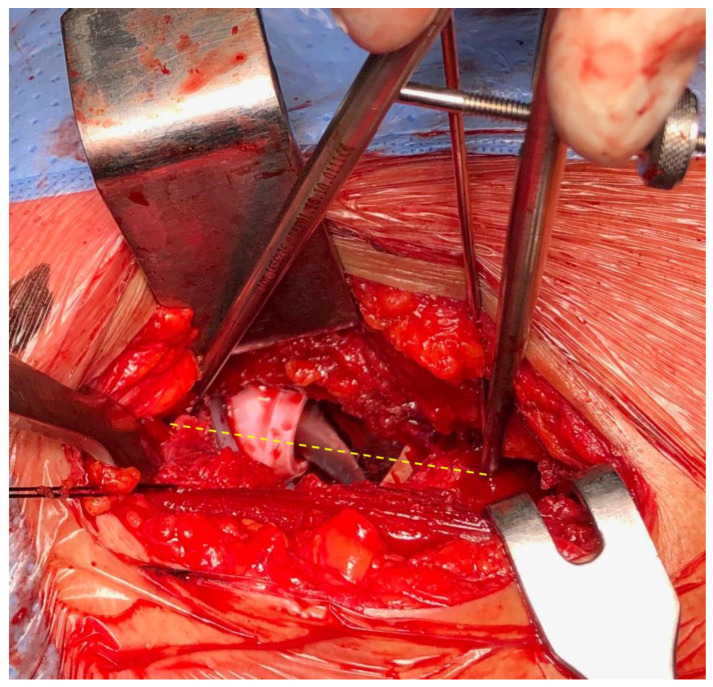
Measurement of the distance between the fixed acetabular and femoral marker (yellow line), with definitive implant.

If satisfactory implant stability and appropriate leg length were achieved, the uncemented femoral component (Amistem H, Medacta International, Castel San Pietro, Switzerland) was implanted. Subsequently, leg length was rechecked using the compass device (Figure 4).

At this stage, adjustments were occasionally feasible using head components of varying measures, always ensuring the stability of the implant. All patients underwent evaluation with anteroposterior radiographs of the pelvis before and after the surgical procedure.

### 2.4. Radiographic Measurements

The LLD was assessed by comparing pre-operative and post-operative radiographs on the same Picture Archiving Communication System (PACS) (Kodak Carestream, Rochester, NY, USA). Positive values of LLD indicated limb lengthening, while negative values indicated shortening. The pre-operative and post-operative LLD were calculated using the trans-teardrop method, as described by Woolson [22], with each data point assessed by two independent observers. 

**Figure 5:**
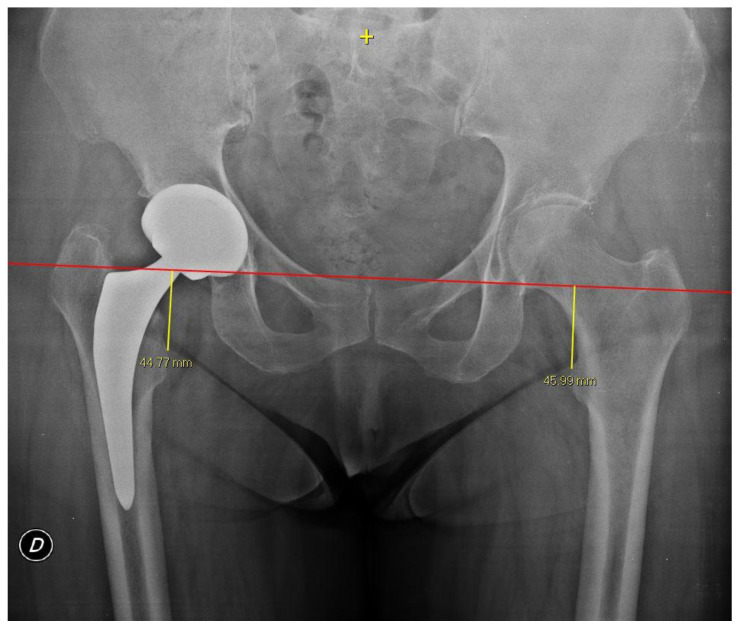
The preoperative and post operative LLD was calculated using the trans-teardrop method, as described by Woolson [22].

Horizontal lines were drawn through the inferior aspect of the teardrop, and the vertical distance between the most prominent point of the lesser trochanter and the trans-teardrop line was measured on both sides [22] (Figure 5). 

After completing these measurements, each dataset was compared using the Bland–Altman plot to evaluate the agreement between the two teardrop measurements made by each independent observer. The Bland–Altman plot depicts the difference between the two quantitative measurements plotted against the averages of the two measurements [23]. 

The mean LLD, standard deviation (SD), and range of values were calculated both pre-operatively and post-operatively as continuous variables. Patients were divided in two groups: (1) post-operative LLD less than 5 mm, and (2) post-operative LLD less than 10 mm. This cutoff was selected based on previous literature, suggesting that LLD less than 5 mm is well tolerated, while between 5 mm and 10 mm is perceived but has no adverse clinical effects [4,24].

## 3. Results

Between September 2017 and December 2018, 35 patients meeting the inclusion criteria underwent THA AMIS procedures in our department. Of these, 18 were females (51.4%) and 17 were males (48.6%), with a mean age of 63.8 years (SD: 10.2; range: 40–82) and mean BMI 26.5 (SD: 3.8; range 19.2–37.9). 

THA was performed on the right side in 20 patients (57.1%) and on the left side in 15 (42.9%). 

Statistical analysis did not reveal any correlation between age, gender, BMI, or side of THA and both pre-operative and post-operative LLD. The mean absolute pre-operative LLD was 3.6 mm (SD: 3.9; range 0.2–19.3), while the mean absolute post-operative LLD was 2.5 mm (SD: 3.0; range 0.0–12.2). Post-operative LLD was less than 5 and 10 mm in 88.2% and 94.1% of the cases, respectively. None of the patients experienced dissatisfaction or required the use of a shoe lift.

**Figure 6:**
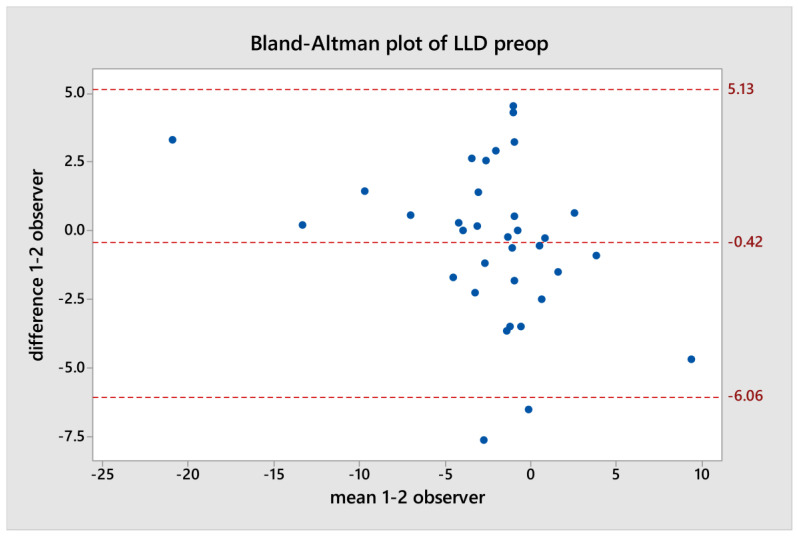
The Bland–Altman plot of preoperative LLD.

**Figure 7:**
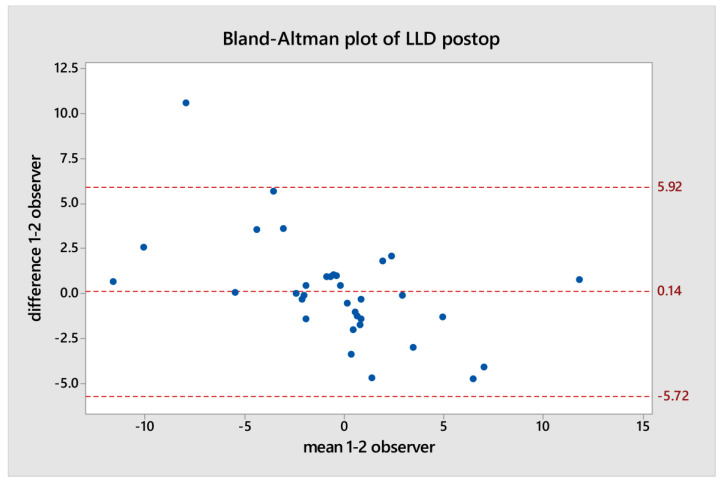
The Bland–Altman plot of postoperative LLD.

Using the Bland–Altman plot, discrepancies were observed in two cases in the pre-operative data and one case in the post-operative data between each LLD measurement of one independent observer and the other (Figure 6 and Figure 7). 

As a result, a good agreement between the measurements was assessed. 

## 4. Discussion

Nowadays, the anterior approach is predominantly utilized as a minimally invasive surgery (MIS), with ample evidence supporting its advantages [25]. Numerous publications have illustrated that the AMIS approach delivers all the benefits expected from a MIS, including shorter rehabilitation, faster return to daily activities, reduced hospital stays [26], diminished post-operative pain [27], minimal blood loss, better short-term functional results [28], and enhanced cosmetic appeal [21,29]. Recent studies revealed no significant difference in component placement between the anterior approach and the various other approaches [30,31,32]. Despite the abundance of literature supporting the efficacy of the AMIS approach, some studies have presented conflicting findings, particularly regarding complications encountered during a surgeon’s initial adoption of the technique [30,33,34,35]. The learning curve affects the results of this technique: surgeons performing fewer than 100 cases via the anterior approach are twice as likely to experience complications [30]. Regardless of surgeon experience, post-operative LLD remains a potential complication. Therefore, an intraoperative method to control limb length during THA performed via the MIS anterior approach could be useful, especially in low-volume hip replacement centers. 

Various methods have been described in the literature to minimize post-operative LLD, which can be divided in four broad categories: pre-operative templating, intra-operative clinical tests, navigation systems, and intra-operative measurement of the distance between two anatomical markers. 

Pre-operative overlay templating remains a standard method to minimize LLD [36], although its reliability in ensuring equal lower limb length is limited due to potential errors in magnification [37]. The use of PACS (Kodak Carestream^®^, Rochester, NY, USA) has facilitated the overlay of conventional plain film radiography [38,39]. In this study, it was preferred to examine all patient cases using anteroposterior digital radiographs of the pelvis on the same PACS, as the mean measured values were found to be accurate and reliable, as demonstrated by Fowler et al. [40]. 

Two radiological techniques are commonly employed in clinical practice to assess LLD. In the methods described by Williamson and Reckling [41], the distance between the most inferior point of the ischia and the lesser trochanter is measured, while in the method described by Woolson et al, the distance between inferior point of the acetabular teardrop and the lesser trochanter is measured [22]. Meermans et al. demonstrated that the teardrop method was more reliable than the bi-ischial line [42], although neither method accounts for hip flexion or abduction deformity at the time of the X-ray, which tends to underestimate the measured LLD, or any causes of LLD that do not involve the hip [43]. Therefore, the use of templates should be complemented with a reliable intra-operative method to achieve better control of leg length.

Intra-operative tests have been developed to assess soft tissue tension and length, such as the shuck test, which was found offer the best correlation with post-operative radiographic LLD [44,45]. However, the intra-operative test has not been scientifically validated, and accurately measuring soft tissue tension during surgery can be challenging due to its reliance on factors such as surgeon experience, type of anesthesia, and the surgical approach [44]. Moreover, the AMIS requires that the patient’s operated leg be positioned on the Mobile Leg Positioner, which is maneuvered outside the surgical field by an expert Product Specialist. Consequently, the surgeon cannot perform a leg-to-leg comparison, and the shuck test is only conducted to assess THA stability. 

Nowadays, intraoperative fluoroscopy remains a reliable method for assessing leg length in real-time without the need for soft tissue tension tests in the AMIS approach [46,47].

Radiological measurement techniques include the transverse rod method to calculate the distance between the ischial tuberosities and the lesser trochanter [48] and overlaying an intra-operative radiograph on top of a preoperative image to see the relative positions of anatomic landmarks [49]. Austin et al. demonstrated that each method effectively minimized LLD, with no statistical difference between them [50]. Certainly, intraoperative imaging enhances surgical precision but requires multiple shots or even live fluoroscopy to achieve proper alignment, potentially leading to longer surgical times and increased radiation exposure for both the patient and surgeon.

Computer-Navigated surgery holds promise in restoring a more normal leg length equality than freehand techniques and eliminating the LLD outliers of greater than 1 cm after THA [51,52,53,54,55]. In the direct anterior approach (DAA), the patient is placed in the supine position, facilitating pelvic tracker placement and the registration process, although there is no statically significant difference in the accuracy or precision of component positioning between the DAA and Posterior Approach groups [51]. Despite precise calculations, the precision largely relies on mapping and referencing points, which are under the surgeon’s control. Moreover, Computer-Navigated surgery does not appear to result in better functioning hip or improved perception of limb length equality, raising questions about the necessity of added time and cost during THA [52,56].

Several intraoperative devices have been developed to assess intraoperative correction of LLD for the lateral and postero-lateral approach [10,11,12,13,14,15,16,17]. McGee and Scott were the first to employ a fine guide wire bent in “U” shape [11]. The use of a large Steinmann pin have been criticized for reliability [12]. Jasty et al. utilized a similar caliper technique [13]. Naito et al. [14], Bose [15], and Shiramizu [16] described techniques employing a Steinman pin and adjustable caliper. Ranawat et al. reported the use of a vertical Steinman pin at the infracotyloid groove of the acetabulum [10]. Mihalko et al. described a technique using a large screw [57]. Matsuda et al. used a ruler [58], while Takigami et al. described another technique using a dual pin retractor [59]. 

While there are approximately 20 different intraoperative techniques described in the literature for achieving limb length equality, none have been utilized during THA performed via the MIS anterior approach, such as the AMIS. Like previously described devices in the literature, the compass device operates on the principle of measuring the distance between a fixed point on the pelvis and a fixed point on the femur. These points were located near to the center of rotation of the hip, specifically on the supero-lateral acetabular edge and on the inter-trochanteric line of femur. As emphasized by Bose [15], it is essential not only to establish stable reference points in both the pelvis and femur but also to accurately reproduce the abduction/adduction position of the femur in space before and after trial component placement to address LLD intra-operatively. Inaccurate femur reposition in terms of abduction or adduction yields greater errors than flexion/extension repositioning. A deviation of 5 to 10 degrees in abduction or adduction can lead in a LLD of 8–17 mm [17]. The supine position and the use of the Leg Positioner alleviate these difficulties related to patient positioning. Conversely, in the lateral decubitus position (e.g., the posterior approach), limb length assessment during surgery relies more on surgeon’s experience than on true anatomical landmarks. The Leg Positioner offers the additional advantage of controlling limb rotation and traction, which is secured on the table. Moreover, in this case series, no complications related to the AMIS Leg Positioner were observed, in contrast to those reported by Matta et al. [49], who employed a different leg positioner. The results of the current study have shown excellent control of LLD with the intraoperative compass device. In this series, 88,2% of patients exhibited a post-operative LLD of less than 5 mm, and 94,1% had a LLD of less 10 mm. The average post-operative LLD was 2.5 mm (SD: 3.0; range 0.0–12.2). This improvement can be attributed to the measurement technique, the utilization of the Leg Positioner, the AMIS technique, adequate soft tissue release to prevent contracture, and the absence of concurrent deformity in the ipsilateral or contralateral limb.

A potential limitation of this technique may arise from inaccurate measurements with the compass device, particularly when encountering a large osteophyte at the superior lip of the acetabulum, difficulty in identifying the inter-trochanteric line of the femur, or variability in vertical position when fixed points were measured. To address this, it is important to remove periacetabular osteophytes to expose the real supero-lateral aspect of the acetabulum and ensure that the point on the intertrochanteric line is as vertical as possible with respect to the mark on the acetabulum. This facilitates the positioning of the compass precisely at the entry points of the K wires. In future studies, it would be beneficial to measure the distance between anatomic markers to evaluate the correlation between intra-operative and the post-operative limb length. Another potential limitation lies in the use of radiographic measurement tools and protocols, which may be susceptible to human error. Typically, measurement errors associated with radiographic methods range from 1 to 3 mm [60]. To mitigate this concern, the trans-teardrop method was preferred due to its demonstrated excellent agreement with leg length measurements in previous studies [61]. Additionally, each dataset, calculated by two independent observers, was evaluated to assess the agreement between the two measurements, utilizing the Bland–Altman plot [23]. Moreover, any cause of LLD involving the contralateral hip or ipsilateral other joints was excluded to minimize the unreliability of the trans-teardrop method [43]. 

The potential use of a CT scan for 3D digital reconstruction could have been beneficial, but it was not performed as it is not part of routine clinical practice and due to ethical considerations regarding unnecessary radiation exposure to subjects. In upcoming research, 3D digital radiography may certainly offer an alternative for obtaining accurate and reliable measurements. This approach could permit the use of software programs capable of accurately calculating x-ray magnification and then adjusting templates accordingly, as reported in published studies [62,63,64]. Another limitation of this study is the small number of patients and the absence of a control group. Consequently, future studies are necessary to enable a comparison in this regard. Such investigations could provide a deeper understanding of the effectiveness and reliability of the techniques employed, potentially leading to advancements in clinical practice.

## 5. Conclusions

In conclusion, the compass device emerges as a valuable tool for ensuring precise limb length control in THA in mini-invasive DAA, offering both efficiency and cost-effectiveness in clinical practice. The main advantages of this technique are its simplicity, accuracy, and predictability. Furthermore, it takes less than five minutes to use, does not require additional surgical incisions or radiation, and offers a favorable benefit–cost ratio for public healthcare facilities.

## Data Availability

The data presented in this study are available on request from the corresponding author due to privacy, legal and ethical reasons.

## References

[1] Enke O., Levy Y.D., Bruce W.J. (2020). Accuracy of Leg Length and Femoral Offset Restoration after Total Hip Arthroplasty with the Utilisation of an Intraoperative Calibration Gauge. Hip Int. J. Clin. Exp. Res. Hip Pathol. Ther..

[2] Gheewala R.A., Young J.R., Villacres Mori B., Lakra A., DiCaprio M.R. (2023). Perioperative Management of Leg-Length Discrepancy in Total Hip Arthroplasty: A Review. Arch. Orthop. Trauma Surg..

[3] Flecher X., Ollivier M., Argenson J.N. (2016). Lower Limb Length and Offset in Total Hip Arthroplasty. Orthop. Traumatol. Surg. Res..

[4] Iwakiri K., Ohta Y., Fujii T., Minoda Y., Kobayashi A., Nakamura H. (2021). Changes in Patient-Perceived Leg Length Discrepancy Following Total Hip Arthroplasty. Eur. J. Orthop. Surg. Traumatol..

[5] Chen G., Nie Y., Xie J., Cao G., Huang Q., Pei F. (2018). Gait Analysis of Leg Length Discrepancy-Differentiated Hip Replacement Patients With Developmental Dysplasia: A Midterm Follow-Up. J. Arthroplasty.

[6] Gupta R., Pathak P., Singh R., Majumdar K.P. (2019). Double-Stitch Technique: A Simple and Effective Method to Minimize Limb Length Discrepancy after Total Hip Arthroplasty. Indian J. Orthop..

[7] Patterson D.C., Grelsamer R.P., Bronson M.J., Moucha C.S. (2017). Lawsuits After Primary and Revision Total Hip Arthroplasties: A Malpractice Claims Analysis. J. Arthroplasty.

[8] Rougereau G., Marty-Diloy T., Langlais T., Pujol N., Boisrenoult P. (2022). Litigation after Primary Total Hip and Knee Arthroplasties in France: Review of Legal Actions over the Past 30 Years. Arch. Orthop. Trauma Surg..

[9] Cunningham D.J., Ryan S.P., Hong C., Mithani S.K., Adams S.B. (2021). Incidence and Risk Factors for Flap Coverage after Total Ankle Arthroplasty. Foot Ankle Int..

[10] Ranawat C.S., Rao R.R., Rodriguez J.A., Bhende H.S. (2001). Correction of Limb-Length Inequality during Total Hip Arthroplasty. J. Arthroplasty.

[11] McGee H.M., Scott J.H. (1985). A Simple Method of Obtaining Equal Leg Length in Total Hip Arthroplasty. Clin. Orthop. Relat. Res..

[12] Huddleston H.D. (1997). An Accurate Method for Measuring Leg Length and Hip Offset in Hip Arthroplasty. Orthopedics.

[13] Jasty M., Webster W., Harris W. (1996). Management of Limb Length Inequality during Total Hip Replacement. Clin. Orthop. Relat. Res..

[14] Naito M., Ogata K., Asayama I. (1999). Intraoperative Limb Length Measurement in Total Hip Arthroplasty. Int. Orthop..

[15] Bose W.J. (2000). Accurate Limb-Length Equalization during Total Hip Arthroplasty. Orthopedics.

[16] Shiramizu K., Naito M., Shitama T., Nakamura Y., Shitama H. (2004). L-Shaped Caliper for Limb Length Measurement during Total Hip Arthroplasty. J. Bone Jt. Surg. Br..

[17] Sarin V.K., Pratt W.R., Bradley G.W. (2005). Accurate Femur Repositioning Is Critical during Intraoperative Total Hip Arthroplasty Length and Offset Assessment. J. Arthroplasty.

[18] Kezer M., Kizilay Y.O. (2024). A Novel Approach for Correcting Limb Length Discrepancy in Total Hip Arthroplasty. Cureus.

[19] Nossa J.M., Muñoz J.M., Riveros E.A., Rueda G., Márquez D., Pérez J. (2018). Leg Length Discrepancy after Total Hip Arthroplasty: Comparison of 3 Intraoperative Measurement Methods. Hip Int. J. Clin. Exp. Res. Hip Pathol. Ther..

[20] Faldini C. (2023). Leg Length Discrepancy after Primary Total Hip Replacement. Musculoskelet. Surg..

[21] Laude F. (2006). Total Hip Arthroplasty through an Anterior Hueter Minimally Invasive Approach. Interact. Surg..

[22] Woolson S.T., Hartford J.M., Sawyer A. (1999). Results of a Method of Leg-Length Equalization for Patients Undergoing Primary Total Hip Replacement. J. Arthroplasty.

[23] Haghayegh S., Kang H.-A., Khoshnevis S., Smolensky M.H., Diller K.R. (2020). A Comprehensive Guideline for Bland-Altman and Intra Class Correlation Calculations to Properly Compare Two Methods of Measurement and Interpret Findings. Physiol. Meas..

[24] O’Brien S., Kernohan G., Fitzpatrick C., Hill J., Beverland D. (2010). Perception of Imposed Leg Length Inequality in Normal Subjects. Hip Int. J. Clin. Exp. Res. Hip Pathol. Ther..

[25] Kucukdurmaz F., Sukeik M., Parvizi J. (2019). A Meta-Analysis Comparing the Direct Anterior with Other Approaches in Primary Total Hip Arthroplasty. Surgeon.

[26] Loh B., Padki A., Yew A., Pang H.N. (2024). Functional Outcome of Direct Anterior versus Posterior Approach in Total Hip Arthroplasty: A Propensity-Matched Asian Study. Singapore Med. J..

[27] Jia F., Guo B., Xu F., Hou Y., Tang X., Huang L. (2019). A Comparison of Clinical, Radiographic and Surgical Outcomes of Total Hip Arthroplasty between Direct Anterior and Posterior Approaches: A Systematic Review and Meta-Analysis. Hip Int. J. Clin. Exp. Res. Hip Pathol. Ther..

[28] Ang J.J.M., Onggo J.R., Stokes C.M., Ambikaipalan A. (2023). Comparing Direct Anterior Approach versus Posterior Approach or Lateral Approach in Total Hip Arthroplasty: A Systematic Review and Meta-Analysis. Eur. J. Orthop. Surg. Traumatol..

[29] Sebečić B., Starešinić M., Culjak V., Japjec M. (2012). Minimally Invasive Hip Arthroplasty: Advantages and Disadvantages. Med. Glas. Off. Publ. Med. Assoc. Zenica-Doboj Canton Bosnia Herzegovina.

[30] den Hartog Y.M., Mathijssen N.M.C., Vehmeijer S.B.W. (2016). The Less Invasive Anterior Approach for Total Hip Arthroplasty: A Comparison to Other Approaches and an Evaluation of the Learning Curve—A Systematic Review. Hip Int. J. Clin. Exp. Res. Hip Pathol. Ther..

[31] Brun O.-C.L., Sund H.N., Nordsletten L., Röhrl S.M., Mjaaland K.E. (2019). Component Placement in Direct Lateral vs Minimally Invasive Anterior Approach in Total Hip Arthroplasty: Radiographic Outcomes From a Prospective Randomized Controlled Trial. J. Arthroplasty.

[32] Luger M., Hochgatterer R., Klotz M.C., Allerstorfer J., Gotterbarm T., Schauer B. (2022). A Single-Surgeon Experience in Reconstruction of Femoro-Acetabular Offset and Implant Positioning in Direct Anterior Approach and Anterolateral MIS Approach with a Curved Short Stem. Arch. Orthop. Trauma Surg..

[33] Müller D.A., Zingg P.O., Dora C. (2014). Anterior Minimally Invasive Approach for Total Hip Replacement: Five-Year Survivorship and Learning Curve. Hip Int. J. Clin. Exp. Res. Hip Pathol. Ther..

[34] Macheras G.A., Christofilopoulos P., Lepetsos P., Leonidou A.O., Anastasopoulos P.P., Galanakos S.P. (2016). Nerve Injuries in Total Hip Arthroplasty with a Mini Invasive Anterior Approach. Hip Int. J. Clin. Exp. Res. Hip Pathol. Ther..

[35] Berndt K., Rahm S., Dora C., Zingg P.O. (2019). Total Hip Arthroplasty with Accolade/Trident through the Direct Minimally Invasive Anterior Approach without Traction Table: Learning Curve and Results after a Minimum of 5 Years. Orthop. Traumatol. Surg. Res..

[36] Vigdorchik J.M., Sharma A.K., Jerabek S.A., Mayman D.J., Sculco P.K. (2021). Templating for Total Hip Arthroplasty in the Modern Age. J. Am. Acad. Orthop. Surg..

[37] Mainard D., Barbier O., Knafo Y., Belleville R., Mainard-Simard L., Gross J.-B. (2017). Accuracy and Reproducibility of Preoperative Three-Dimensional Planning for Total Hip Arthroplasty Using Biplanar Low-Dose Radiographs: A Pilot Study. Orthop. Traumatol. Surg. Res..

[38] Lim Y.W., Chang Y.J., Kwon S.Y., Kim Y.S. (2013). A Simple Method Using a PACS to Minimize Leg Length Discrepancy in Primary THA: A Method to Minimize Leg Length Discrepancy. J. Arthroplasty.

[39] Pongkunakorn A., Udomluck P., Aksornthung C., Wangjiraphan N. (2023). Digital Templating of THA Using PACS and an IPhone or IPad Is as Accurate as Commercial Digital Templating Software. Clin. Orthop. Relat. Res..

[40] Fowler J.R., Ilyas A.M. (2011). The Accuracy of Digital Radiography in Orthopaedic Applications. Clin. Orthop. Relat. Res..

[41] Williamson J.A., Reckling F.W. (1978). Limb Length Discrepancy and Related Problems Following Total Hip Joint Replacement. Clin. Orthop. Relat. Res..

[42] Meermans G., Malik A., Witt J., Haddad F. (2011). Preoperative Radiographic Assessment of Limb-Length Discrepancy in Total Hip Arthroplasty. Clin. Orthop. Relat. Res..

[43] Guo R., Chen J.Y., Zhang G., Zhou Y., Chen J., Chai W. (2019). Calculation Method to Predict Postoperative Limb Length in Patients Undergoing THA Following Developmental Dysplasia of Hips. BMC Musculoskelet. Disord..

[44] Lecoanet P., Vargas M., Pallaro J., Thelen T., Ribes C., Fabre T. (2018). Leg Length Discrepancy after Total Hip Arthroplasty: Can Leg Length Be Satisfactorily Controlled via Anterior Approach without a Traction Table? Evaluation in 56 Patients with EOS 3D. Orthop. Traumatol. Surg. Res..

[45] Takao M., Nishii T., Sakai T., Sugano N. (2016). Postoperative Limb-Offset Discrepancy Notably Affects Soft-Tissue Tension in Total Hip Arthroplasty. J. Bone Jt. Surg. Am..

[46] Ji W., Stewart N. (2016). Fluoroscopy Assessment during Anterior Minimally Invasive Hip Replacement Is More Accurate than with the Posterior Approach. Int. Orthop..

[47] Bingham J.S., Spangehl M.J., Hines J.T., Taunton M.J., Schwartz A.J. (2018). Does Intraoperative Fluoroscopy Improve Limb-Length Discrepancy and Acetabular Component Positioning During Direct Anterior Total Hip Arthroplasty?. J. Arthroplast..

[48] Nam D., Sculco P.K., Abdel M.P., Alexiades M.M., Figgie M.P., Mayman D.J. (2013). Leg-Length Inequalities Following THA Based on Surgical Technique. Orthopedics.

[49] Matta J.M., Shahrdar C., Ferguson T. (2005). Single-Incision Anterior Approach for Total Hip Arthroplasty on an Orthopaedic Table. Clin. Orthop. Relat. Res..

[50] Austin D.C., Dempsey B.E., Kunkel S.T., Torchia M.T., Jevsevar D.S. (2019). A Comparison of Radiographic Leg-Length and Offset Discrepancies between 2 Intraoperative Measurement Techniques in Anterior Total Hip Arthroplasty. Arthroplast. Today.

[51] Bradley M.P., Benson J.R., Muir J.M. (2019). Accuracy of Acetabular Component Positioning Using Computer-Assisted Navigation in Direct Anterior Total Hip Arthroplasty. Cureus.

[52] Rajpaul J., Rasool M.N. (2018). Leg Length Correction in Computer Assisted Primary Total Hip Arthroplasty: A Collective Review of the Literature. J. Orthop..

[53] Ellapparadja P., Mahajan V., Atiya S., Sankar B., Deep K. (2016). Leg Length Discrepancy in Computer Navigated Total Hip Arthroplasty—How Accurate Are We?. Hip Int. J. Clin. Exp. Res. Hip Pathol. Ther..

[54] Laggner R., Oktarina A., Windhager R., Bostrom M.P.G. (2023). Changes in Leg Length and Hip Offset in Navigated Imageless vs. Conventional Total Hip Arthroplasty. Sci. Rep..

[55] Dundon J.M., Mays R.R. (2019). Revising Substantial Leg Length Discrepancy in Total Hip Arthroplasty Using Computer-Assisted Navigated Systems: A Case Series of Three Patients. Cureus.

[56] Hecht C.J., Nedder V.J., Porto J.R., Morgan K.A., Kamath A.F. (2024). Are Robotic-Assisted and Computer-Navigated Total Hip Arthroplasty Associated with Superior Outcomes in Patients Who Have Hip Dysplasia?. J. Orthop..

[57] Mihalko W.M., Phillips M.J., Krackow K.A. (2001). Acute Sciatic and Femoral Neuritis Following Total Hip Arthroplasty. A Case Report. J. Bone Jt. Surg. Am..

[58] Matsuda K., Nakamura S., Matsushita T. (2006). A Simple Method to Minimize Limb-Length Discrepancy after Hip Arthroplasty. Acta Orthop..

[59] Takigami I., Itokazu M., Itoh Y., Matsumoto K., Yamamoto T., Shimizu K. (2008). Limb-Length Measurement in Total Hip Arthroplasty Using a Calipers Dual Pin Retractor. Bull. NYU Hosp. Jt. Dis..

[60] Sariali E., Mauprivez R., Khiami F., Pascal-Mousselard H., Catonné Y. (2012). Accuracy of the Preoperative Planning for Cementless Total Hip Arthroplasty. A Randomised Comparison between Three-Dimensional Computerised Planning and Conventional Templating. Orthop. Traumatol. Surg. Res..

[61] Sykes A., Hill J., Orr J., Humphreys P., Rooney A., Morrow E., Beverland D. (2015). Patients’ Perception of Leg Length Discrepancy Post Total Hip Arthroplasty. Hip Int. J. Clin. Exp. Res. Hip Pathol. Ther..

[62] Knafo Y., Houfani F., Zaharia B., Egrise F., Clerc-Urmès I., Mainard D. (2019). Value of 3D Preoperative Planning for Primary Total Hip Arthroplasty Based on Biplanar Weightbearing Radiographs. Biomed Res. Int..

[63] Mancino F., Fontalis A., Magan A., Plastow R., Haddad F.S. (2024). The Value of Computed Tomography Scan in Three-Dimensional Planning and Intraoperative Navigation in Primary Total Hip Arthroplasty. Hip Pelvis.

[64] Moralidou M., Di Laura A., Henckel J., Hothi H., Hart A.J. (2020). Three-Dimensional Pre-Operative Planning of Primary Hip Arthroplasty: A Systematic Literature Review. EFORT Open Rev..

